# Correlation between ankle‐brachial index and subtle cognitive decline

**DOI:** 10.1002/brb3.3019

**Published:** 2023-04-23

**Authors:** Hui‐Feng Guo, Yi Wu, Guo‐Xiang Fu, Jie Li, Jie‐Hua Zhu

**Affiliations:** ^1^ Department of Gerontology Shanghai Jiao Tong University Affiliated Sixth People's Hospital Shanghai P. R. China; ^2^ The International Peace Maternity and Child Health Hospital, School of Medicine Shanghai Jiao Tong University Shanghai P. R. China; ^3^ Shanghai Tenth People's Hospital Tongji University Shanghai P. R. China

**Keywords:** Alzheimer's disease, ankle‐brachial index, correlation, subtle cognitive decline

## Abstract

**Background:**

Subtle cognitive decline (SCD) is considered the early stage of Alzheimer's disease (AD) and is of great clinical significance for the prevention and treatment of AD. The ankle‐brachial index (ABI) has been reported to be associated with cognitive impairment; however, there are few studies on the relationship between ABI and SCD.

**Methods:**

From August 2019 to April 2021, subjects were recruited to participate in a cognitive function test at the Shanghai Sixth People's Hospital. Based on the test results, 217 patients with SCD were selected as the experimental group and 259 patients with normal cognitive function were selected as the control group. The data of the two groups were compared, and the correlation between the ABI and cognitive decline was analyzed.

**Results:**

There were significant differences in age, sex, smoking status, hypertension, diabetes, triglycerides, serum creatinine, and ABI (*p* < .05)between the two groups. Logistic regression analysis showed that age, hypertension, diabetes, and ABI influenced cognitive decline(*p* < .05). After correcting for other factors, ABI was independently related to cognitive decline. Pearson's correlation analysis showed that a low ABI (<0.9) had a significant effect on memory and visual space of the cognitive domain (*p* < . 05).

**Conclusions:**

ABI is significantly associated with SCD and may be a critical tool to predict early cognitive decline.

## Graphical abstract

A total of 217 cases with subtle cognitive decline were selected as the experimental group and 259 cases with normal cognitive test as the control group. The data of the two groups were compared, and the correlation between ABI and cognitive decline was analyzed. The results indicate ABI is associated with subtle cognitive decline and may be used to predict early cognitive decline.

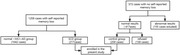

## 1 INTRODUCTION

Dementia is a disease that seriously affects human health and places a heavy burden on patients, their families, and society (Ferri et al., 2005). With the process of aging, the incidence of dementia is steadily increasing (GBD 2016 Neurology Collaborators, 2019). The leading cause of dementia is Alzheimer's disease (AD), which is predicted to have a prevalence of one in two to three people over the age of 85 by 2030 (Brody, 2011; Brookmeyer et al., 2007; Prince et al., 2013). At present, although some progress has been made in the treatment of AD, the therapeutic effect is still far from satisfactory (McDade & Bateman, 2017; Sperling et al., 2011a). Therefore, this research focuses on early identification and intervention of AD (Edmonds et al., 2015; Jack et al., 2010; Vellas et al., 2011). According to the National Institute on Aging and the Alzheimer's Association (NIA‐AA), AD is divided into following three stages (Jack et al., 2013, Sperling et al., 2011b): AD preclinical stage (subtle cognitive decline (SCD), AD‐derived mild cognitive impairment (MCI), and AD dementia stage. In the stage ofMCI and AD, irreversible neurodegenerative diseases and neuronal damage have occurred, and the effect of intervention has not been satisfactory (Iadecola et al., 2019; Sperling et al., 2011). Recent studies have shown that about 30% of AD risk factors can be prevented through early detection and timely intervention (Jack et al., 2018; Papp et al., 2020). As an early stage of AD, SCD research has become one of the hotspots of AD research (Edmonds et al., 2015; Thomas et al., 2020) . The methods of testing cognitive function include cognitive scale, gene detection, imaging, and cerebrospinal fluid (CSF) biomarkers. Standardized cognitive scale testing needs specially trained personnel and special sites. Magnetic resonance imaging (MRI), gene detection, and CSF testing need expensive equipment, and some tests are traumatic, so they are not suitable for large‐scale clinical application. Looking for a simple marker that can predict cognitive dysfunction may help clinicians provide timely interventions and reduce the burden caused by cognitive impairment.

Ankle‐brachial index (ABI) refers to the ratio of ankle artery blood pressure to brachial artery blood pressure by measuring the systolic blood pressure of the posterior tibial artery or anterior tibial artery and the brachial artery (Aboyans et al., 2012). ABI is convenient and sensitive and is mainly used to detect early‐stage peripheral arterial diseases of the lower extremities (Criqui et al., 1992). Subsequent studies have shown that a decrease in the ABI is an independent risk factor for cardio‐cerebrovascular events and a strong predictor of total mortality and cardiovascular mortality (Bush et al., 2009; Heald et al., 2006). At present, the ABI can be used not only for the diagnosis of peripheral arterial diseases of the lower extremities but also for the risk stratification of diseases, such as atherosclerotic diseases, which has important clinical applications. There have been reports on the correlation between the ABI and cognitive function (Desormais et al., 2018; Hilal et al., 2014; Tarraf et al., 2018); however, there are few reports on the association between the ABI and SCD. We used a standardized neuropsychological scale to screen SCD personnel and measured ABI at the same time to analyze whether there is a correlation between ABI and SCD and whether it can be used as an indicator of early cognitive decline screening.

## 2 Materials AND METHODS

### 2.1 Participants

**FIGURE 1:**
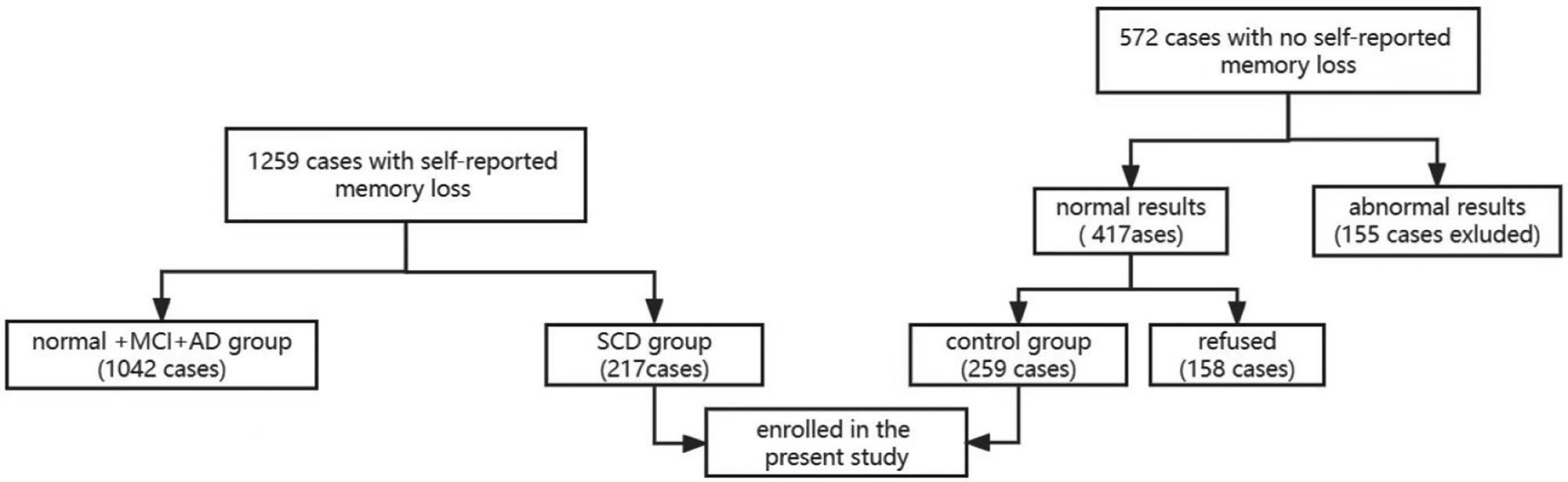
Schematic of the recruitment and enrollment of volunteers.

From August 2019 to April 2021, individuals over 50 years of age who experienced memory loss were recruited at the Cognitive Clinic of Shanghai's Sixth People's Hospital. After obtaining informed consent, these individuals were tested by a neuropsychological scale, a head MRI, related laboratory examinations, and an ABI. A total of 1259 people were divided into normal cognitive function, SCD, MCI, and AD groups according to their test results, with the SCD group designated as the experimental group. Concurrently, 572 volunteers who had no memory loss were recruited in the outpatient clinic for scale testing; participants with a normal scale test were selected as the normal control group, and the above examinations were carried out in this control group with the consent of the participants and their families. The inclusion criteria were as follows: age more than 50 years; primary school education and above; and normal hearing and eyesight. The exclusion criteria were: individuals diagnosed with MCI and AD through testing; individuals with a history of cerebrovascular diseases, such as cerebral infarction, brain trauma, brain tumor, Parkinson's disease, cerebral epilepsy, psychosis, and dysplasia; individuals with a Hamilton depression scale (17 items) score greater than 12(Hamilton, 1960); individuals with other cognitive diseases, such as hypothyroidism, folic acid or B12 deficiency, alcohol abuse, drug abuse, syphilis and AIDS; individuals with a more than 20 mmHg blood pressure difference between their two upper arms; and individuals with an ABI greater than 1.4. After excluding those who did not meet the criteria, 476 individuals were enrolled in the study including 259 in the control group (NC group) and 217 in the experimental group (SCD group). A flowchart of case enrollment is shown in Figure 1. All the participants signed an informed consent form and the study was approved by the Ethics Committee of the Shanghai Sixth People's Hospital.

### 2.2 Neuropsychological assessment

The cognitive scale test was conducted by professionally trained assessors in a specialized neuropsychological room. Those who participated in the scale evaluation did not participate in judging cognitive function. All participants underwent strict scale tests to accurately evaluate their memory, language, space, attention, execution, social cognition, and other functions, including a Mini‐mental State Examination (MMSE), a Montreal cognitive assessment (Chinese version of MOCA) (Lu et al., 2011), a Hamilton Depression Scale, an Auditory Verbal Learning Test (AVLT) (Guo et al., 2001), a Boston Naming Test (BNT) (Guo et al., 2006), a Symbol Digit Modalities Test (SDMT) (Guo et al., 2005), a Complex Figure Test (CFT) (Zhao et al., 2013), and a Trail Making Test‐A and ‐B (TMT‐A, TMT‐B) (Zhao et al., 2007) .

### 2.3 ABI determination

The participants were placed in a supine position and made to rest for 10 min after which a cuff of an appropriate size was used to measure blood pressure with a handheld Doppler stethoscope (VP‐1000, Omron, Japan). The testers were trained to ensure their accuracy. The ratio of the high systolic blood pressure of the bilateral dorsalis pedis artery and the posterior tibial artery divided by the high systolic blood pressure of the bilateral brachial artery was defined as the ABI. We compared the ABI of the left and right sides of each participant and considered the lower one to be the participant's ABI. We defined a normal ABI as 0.9 (Ko & Bandyk, 2013). Previous reports state that an ABI greater than 1.4 indicates incompressible and severely calcified blood vessels, resulting in an abnormal rise in blood pressure (Aboyans et al., 2012; Saskia et al., 2008); therefore, such individuals were not included in our study.

### 2.4 Biochemical index

Venous blood samples were collected after participants had fasted for 8 h, and were immediately sent for examination to determine blood glucose, blood lipids, folic acids, vitamin B12, serum creatinine, serum uric acid, and thyroid function. Postprandial blood glucose levels were measured 2 h after eating.

### 2.5 Diagnostic criteria

The diagnosis of SCD according to the Jak/Bondi criteria is: impaired scores (defined as an 1SD below the age‐corrected normative mean) on two of the six neuropsychological measures in different cognitive domains (Edmonds et al., 2015).

### 2.6 Statistical analyses

Statistical analyses were performed using SPSS software version 24.0 (IBM Corp., Armonk, NY). The data were expressed as means ± standard deviations ($\bar{x}\pm s$), and the *t*‐test was used for comparisons between groups. The chi‐square test was used to compare counting data between groups. The risk factors for cognitive impairment were analyzed by binary logistic regression using logistic regression analysis to study the relationship between low ABIs and SCD. Furthermore, the correlation between participant's cognitive domain scores and their ABI was analyzed using the Pearson correlation analysis. Data with a *p* value less than .05 were considered statistically significant.

## 3 RESULTS

The general characteristics of the study participants are presented in Table 1. Kolmogorov–Smirnov test (KS test) was used to test the normality of ABI, the results of *p* > .05 indicated that the data were of normal distribution. There were significant differences in age, sex, smoking history, prevalence rates of hypertension and diabetes, fasting blood glucose, postprandial blood glucose, triglycerides, serum creatinine, and ABI between the two groups, but there were no significant differences in the other indexes.

**TABLE 1:** General characteristics of study participants

Index	NC (n = 259)	SCD (n = 217)	*p* value
Age, years	63.15 ± 9.24.	64.59 ± 10.32	.017
Sex (male,%)	172 (66.41%)	134 (61.75%)	.039
Education, years	12.29 ± 3.54	12.03 ± 2.91	.527
BMI (kg/m^2^)	23.17 ± 3.14	23.51 ± 3.39	.231
Smoking history	43(16.60%)	42(19.357%)	.025
Drinking (n, %)	71 (27.41%)	62 (28.57%)	.573
Hypertension (n, %)	61 (23.55%)	59 (27.19%)	.041
Diabetes (n, %)	22 (8.49%)	29 (11.20%)	.005
CAD (n, %)	29 (11.20%)	33 (15.21%)	.032
FBG (mmol/L)	5.27 ± 0.89	5.97 ± 1.82	.003
PBG (mmol/L)	8.32 ± 2.35	9.31 ± 3.52	.009
Scr (μmol/L)	81.35 ± 20.39	83.24 ± 24.37	.037
Homocysteine (μmol/L)	16.35 ± 4.32	17.02 ± 5.13	.405
TC (mmol/L)	4.17 ± 1.09	3.97 ± 0.93	.271
TG (mmol/L)	1.18 ± 0.72	1.39 ± 0.81	.032
HDL‐C (mmol/L)	1.19 ± 0.41	1.17 ± 0.38	.325
LDL‐C (mmol/L)	2.15 ± 0.79	2.09 ± 0.73	.251
ABI	1.14 ± 0.30	1.02 ± 0.37	.009
ABI ≤ 0.9(n, %)	35(13.51%)	49(22.58%)	.007

ABI, ankle‐brachial index; CAD, coronary artery disease; FBG, fasting blood glucose; HDL‐C, high‐density lipoprotein cholesterol; LDL‐C, low‐density lipoprotein cholesterol; MI, body mass index; NC, normal control; PBG, postprandial blood glucose; SCD, subtle cognitive decline; Scr, serum creatinine; Smoking history, meant a history of smoking more than 10 years; TC, total cholesterol; TG, triglycerides;

Participants conducted MRI examinations (due to financial and other reasons, functional magnetic resonance imaging was not performed), and there was no significant difference in indicators such as brain atrophy and subcortical white matter lesions between the two groups.

The scale scores of the two groups are shown in Table 2. There were significant differences in the memory, visual space, MMSE, and MOCA scores between the two groups, the SCD group was significantly lower than the control group. The AVLT score in the memory test was mainly for delayed memory, which decreased significantly.

**TABLE 2:** Scale scores of the NC and SCD groups

Index	NC(n = 259)	SCD (n = 217)	F (*p*)
MMSE	28.7 ± 1.9	26.2 ± 1.8	49.527 (<.001)
MOCA	25.3 ± 3.1	22.4 ± 3.6	63.419 (<.001)
AVLT recognition	21.4 ± 4.3	20.19 ± 3.9	1.823 (.082)
AVLT delayed recall	6.3 ± 1.8	5.2 ± 1.9	6.879 (.029)
BNT	23.9 ± 4.2	22.8 ± 3.6	0.302 (.257)
SDMT	38.6 ± 11.4	37.7 ± 11.9	0.219 (.324)
TMT‐A	52.7 ± 25.3	55. 3 ± 26.9	0.182 (.307)
TMT‐B	131.9 ± 42.1	138.5 ± 37.5	0.327 (.165)
Rey CFT copy	34.2 ± 3.1	31.5 ± 4.4	8.725 (.021)
Rey CFT recall	16.6 ± 6.5	13.2 ± 6.9	8.957 (.012)

AVLT, Auditory‐Verbal Learning Test; BNT, Boston Naming Test; MoCA, Montreal Cognitive Assessment; MMSE, Mini‐Mental State Examination; NC, normal control; Rey CFT, Rey‐Osterrieth Complex; SCD, subtle cognitive decline; SDMT, Symbol Digit Modality Test; TMT‐A, TMT‐B, Trail Making Test Parts A and B;

Taking cognitive impairment as the dependent variable and age, sex, smoking history, prevalence rates of hypertension and diabetes, triglycerides, serum creatinine, and ABI as independent variables, a binary logistic regression analysis was carried out. The results showed that age, hypertension, diabetes, and ABI had a significant effect on the decline of cognitive function (Table 3). Logistic regression analysis showed that after adjusting for age, sex, smoking, hypertension, diabetes, triglycerides, serum creatinine, and other factors, the ABI remained an independent risk factor for cognitive decline (Table 4).

**TABLE 3:** Binary logistic regression analysis of factors influencing cognitive function

Index	*Β*	SE	Wald	*p* value	OR(95% CI)
Age	1.415	0.573	9.732	.036	2.915 (1.338–4.527)
Sex	0.022	0.043	0.594	.341	1.201 (0.875–1.153)
Smoking history	0.054	0.039	1.877	.216	1.183 (0.912–1.952)
TG	0.033	0.159	0.712	.347	1.591 (1.174–2.381)
Hypertension	2.537	0.962	11.394	.015	3.579 (1.472–7.894)
Diabetes	0.142	0.061	4.927	.029	1.214 (1.034–1.153)
CAD	0.019	0.023	1.317	.195	1.103 (0.951–1.192)
Scr	0.152	0.203	0.632	.472	0.952 (0.685–1.127)
ABI	3.481	1.472	17.547	.000	4.259 (2.158–9.215)

ABI, ankle‐brachial index; CAD, coronary artery disease; CI, confidence interval; OR, odds ratio; Scr, serum creatinine; SE, standard error; TG, triglycerides.

**TABLE 4:** Logistic regression analysis on the relationship of low ankle‐brachial index (≤0.9) with subtle cognitive decline

	OR	95% CI	*p* value
Model 1	3.715	2.391–7.528	.003
Model 2	3.249	2.065–6.935	.021
Model 3	2.956	1.872–6.459	.037

Model 1 is adjusted for smoking history.

Model 2 is adjusted for smoking history, age, and sex.

Model 3 is adjusted for smoking history, age, sex, hypertension, diabetes, triglycerides, and serum creatinine.

The correlation analyses between the results of each cognitive domain scale and the ABI are shown in Table 5. It can be seen that a decrease in ABI affects the functions of memory and visual space in the cognitive domain.

**TABLE 5:** Correlation between the scores of different cognitive domains and low ankle‐brachial index (ABI≤0.9)

Index	ABI	
	*r*	*p* value
Memory	0.391	.006
Language	0.185	.201
Attention	0.092	.387
Visuospatial ability	0.509	.000
Executive function,	0.152	.347
Social cognition	0.142	.561

## 4 DISCUSSION

SCD was first proposed by Reisberg et al. (1982, 1986) and he further pointed out that this group of people with subjective memory impairment appeared before the onset of MCI and AD symptoms. Jessen et al. (2014) proposed a diagnostic framework: SCD means that patients mainly show memory loss, but objective examination does not reach the stage of mild cognitive impairment or dementia. This cognitive decline is continuous. It was not related to other diseases, accidents, or acute events. SCD is associated with a high incidence and risk of conversion to mild MCI and AD; hence, SCD is considered a preclinical stage of AD. Patients with SCD are significantly more likely to develop MCI and dementia than those without SCD (Jack et al., 2018; Mitchell et al., 2014). β‐amyloid protein, tau protein, structural or functional MRI, and even fluorodeoxyglucose positron emission tomography computed tomography (FDG‐PET) and Pittsburgh B amyloid positron emission tomography (PIB‐PET) in CSF have been reported to confirm the existence of AD‐related pathological changes in SCD (Braak et al., 2011; Kapasi et al., 2017; Thomas et al., 2020). Therefore, SCD is an important predictor of dementia. Effective intervention in patients with SCD is of great significance for delaying the course of the disease, preventing the occurrence of dementia, improving quality of life, and relieving family or social pressure. However, CSF, MRI, and other examinations cannot be performed on a large scale because of their traumatic and expensive nature; hence, they are not suitable for screening SCD in the general population. Thus, most cognitive decline diagnoses are completed using the scale mentioned in this study.

However, as the symptoms of SCD are not obvious, they do not have a significant impact on daily life and are often ignored. By the time patients visit a doctor for a significant decline in memory, cognitive function is often impaired, with the disease having progressed to MCI or AD. Even if patients visit a doctor before this, a considerable number of medical institutions do not have professional‐scale testing personnel, let alone MRI and CSF examinations.

The ABI reflects hardening of the peripheral artery and is a sign of arteriosclerosis (Criqui et al., 1992). Early‐stage arteriosclerosis is one of the diagnostic criteria for peripheral arterial disease. Moreover, an ABI of less than or equal to 0.9 is an index for peripheral arterial disease with high sensitivity and specificity (Ko & Bandyk, 2013). Consequently, peripheral arterial disease is a risk factor for atherosclerosis in other vascular systems (Bush et al., 2009; Heald et al., 2006). Cognitive dysfunction is a disease involving many factors, with complex pathophysiological processes, and vascular abnormalities are one of its causes (Kovacic & Fuster, 2012; Rabkin, 2012).

The results of our study showed that with increasing age, the cognitive scale score and the incidence of dementia in the group with an ABI of less than or equal to 0.9 were significantly higher than those in the group with a normal ABI. This correlates with previous studies reporting that a low ABI is associated with cognitive impairment and can be used as an independent predictor of cognitive impairment (Desormais et al., 2018; Hilal et al., 2014; Tarraf et al., 2018).

In this study, 1831 people were tested using the scale test and 476 were enrolled in the experimental group. The control group consisted of individuals with no memory loss. Hence, to ensure this, those who complained of memory loss were not included in the control group even if their scale test was normal.

We used the standard cognitive scale for testing, screened personnel with the SCD, and analyzed the relationship between the ABI and cognitive function. We found that people who joined the SCD group had significantly smaller ABIs than the NC group. The scale test showed that even at the SCD stage, a decrease in ABI was significantly correlated with a decline in cognitive function, which was independent of other risk factors for cognitive function such as age and blood glucose levels. Furthermore, the results showed that the decrease in ABI mainly affected memory and visual‐spatial abilities, whereas there was no significant difference in other aspects of the cognitive domain between the two groups in the study. This differs from previous reports (Rabin et al., 2014; Rabkin, 2012; Thomas et al., 2020), which may be due to different admission criteria. We chose the SCD population as the experimental group, while previous studies selected the MCI or AD populations. In addition, there were no significant differences between the sexes in our study.

ABI is an indicator of atherosclerosis. The mechanism underlying the effect of ABI on cognitive function may be that ABI reflects the degree of systemic atherosclerosis. Hence, a decrease in the ABI may indicate hardening of intracranial arteries and cerebrovascular disease, which may in turn lead to the decreased rate of amyloid β (A β) clearance and the deformation of neurons. Furthermore, decreased arterial elasticity results in a decreased buffering capacity of large arteries to high arterial pressures, leading to structural remodeling, functional changes of arterioles, and decreased cerebral perfusion, resulting in white matter lesions and destruction of the connection between cortical gray matter structures. Vascular sclerosis is important as it leads to collateral circulation decompensation, hypoperfusion, and so on (Hazzouri & Yaffe, 2014; O'Rourke & Safar, 2005; Shindo et al., 2016; Xie et al., 2020; Zeki et al., 2014).

Previous studies have shown that amyloid deposition and brain atrophy can be observed early in cognitive decline (Calcet et al., 2022; Cersonsky et al., 2022). In this study, there was no significant difference in the MRI results between the two groups, which might be because we did not perform functional MRI and PET examinations. The small sample size may also be the reason for no significant difference between the two groups.

The limitations of this study are that the participants were not randomly screened from the general population, the number of cases in each group was small, and genetic and CSF tests were not performed. Additionally, participants should be followed‐up to further verify the results of this study and analyze the proportion of people with different ABIs who developed AD.

In conclusion, our results indicate that the ABI may be used as a predictive indicator of SCD to judge early decline in cognitive function and take timely interventions. ABI detection is convenient and inexpensive; therefore, it can be used as a tool for large‐scale epidemiological investigations of cognitive impairment. This finding has important clinical significance in the prediction of early cognitive impairment in this population, and hence, timely intervention to delay progression can reduce the burden of AD for patients, their families, and society.

### PEER REVIEW

The peer review history for this article is available at https://publons.com/publon/10.1002/brb3.3019.

## Data Availability

The datasets used and/or analyzed during the current study are available from the corresponding author on reasonable request.
